# An 85-year-old woman with Miller Fisher syndrome

**DOI:** 10.12669/pjms.295.3793

**Published:** 2013

**Authors:** Shu-hui Wang, Yong-bo Zhang, Yan-chen Xie, De-xin Wang, Ji-mei Li

**Affiliations:** 1 Shu-hui Wang, Department of Neurology, Beijing Friendship Hospital, Capital Medical University, Beijing, China.; 2 Yong-bo Zhang, Department of Neurology, Beijing Friendship Hospital, Capital Medical University, Beijing, China.; 3 Yan-chen Xie, Department of Neurology, Beijing Friendship Hospital, Capital Medical University, Beijing, China.; 4 De-xin Wang, Department of Neurology, Beijing Friendship Hospital, Capital Medical University, Beijing, China.; 5 Ji-mei Li, Department of Neurology, Beijing Friendship Hospital, Capital Medical University, Beijing, China.

**Keywords:** Miller Fisher’s syndrome, Aged, 80 and over, Ophthalmoplegia

## Abstract

Miller Fisher’s syndrome (MFS) commonly presents in the fourth and fifth decades and are rare in people over 70 years. An 85-year-old female with no significant medical history presented with upper extremity anesthesia, ptosis, and unsteady gait. The patient had a history of hypertension and diabetes mellitus. Physical examination showed bilateral total external ophthalmoplegia, areflexia, and cerebellar ataxia. Radiological and laboratory studies were unremarkable. Lumbar puncture showed albuminocytological dissociation. The combined history, physical examination, and lumbar puncture results established a presumptive diagnosis of MFS. Intravenous immunoglobulin was given for 5 days. The patient gradually improved 10 days after the onset of symptoms. Ophthalmoplegia had fully recovered after 6 months. To the best of our knowledge, this case represented the oldest patient with MFS.

## INTRODUCTION

Miller Fisher’s syndrome (MFS), which was originally described in 1956, is characterized by the triad of external ophthalmoplegia, cerebellar ataxia, and the absence of tendon reﬂexes. It is considered a variant form of Guillain-Barré syndrome (GBS) because half of patients with MFS eventually experience profound weakness. MFS is a relatively rare disorder and generally presents in the fourth and fifth decades. The syndrome is uncommon in the very elderly. Thus, the authors report a case of an elderly patient who has suffered from MFS at 85 years of age.

## CASE REPORT

Four days before admission, an 85 years old woman developed mild anaesthesia in her hands after an upper respiratory tract infection, and she was afebrile. Over the next two days, she developed difficulty in opening her eyes and could not walk independently because of unsteady gait. She was then admitted to hospital. The patient had a history of hypertension and diabetes mellitus and received antihypertensive medicine and insulin therapy. She had not been exposed to neurotoxic substances.

Physical examination revealed normal cardiovascular and respiratory function. Examination of the cranial nerves showed bilateral blepharoptosis with total external ophthalmoplegia. The eyeballs were ﬁxed in the central position, and there was no eye movement in any direction. Both pupils were dilated and the pupillary light reflexes were absent. There was distal predominant weakness in all four limbs of Medical Research Council (MRC) grade 4. All the tendon reflexes were absent. Ataxia was observed in the upper and lower limbs, with similar severity. Plantar response was flexor bilaterally. There were no sensory deficits for any modality.

Routine laboratory tests were normal. Cerebrospinal ﬂuid (CSF) obtained on the following day after admission to hospital showed an increased protein level of 76.9 mg/dl and no white cells. No organisms were cultured. Analyses of the CSF for Coxsackie, cytomegalovirus, Epstein–Barr virus, herpes simplex virus serologies were negative. Serum CA125, CEA and AFP were in normal range. Chest radiograph and electromyography (EMG) were normal. Computed tomography and magnetic resonance imaging of the brain revealed mild generalized atrophy and multiple old lacunar infarctions within the basal ganglia bilaterally. 

On the basis of the neurological findings, a clinical diagnosis of MFS was established. Intravenous immunoglobulin was given for 5 days at a dose of 0.4g/kg/day.

The patient gradually improved 10 days after the onset of symptoms. Her eye movements were partially improved and she was able to walk several meters. She was discharged at 14 days after admission. When reviewed in the clinic 6 months after discharge, her eye symptoms had fully recovered. Followed up by telephone at two years after onset, the symptoms had not recurred.

## DISCUSSION

In 1956 Miller Fisher described three patients of an acute neurological illness characterized by ophthalmoplegia, ataxia, and areﬂexia. This illness is referred to as the MFS and is considered an unusual variant of GBS, because about half of patients who present with MFS progress to GBS. In MFS, most patients initially have ophthalmoplegia followed by gait and limb ataxia. Other cranial nerves, most notably the facial nerves, may also be affected. Motor strength is usually preserved. Most patients have a preceding respiratory tract infection in MFS. The disease generally follows a benign course with complete resolution in the majority of patients over an average period of 10 weeks. According to the diagnostic criteria put forward by Odaka, this patient was diagnosed as MFS.

The reported incidence of MFS has been estimated at 0.09 cases per 100,000 persons per year, though no large epidemiological studies on MFS have been published. Children and adults of all ages can be affected by MFS. In a recent study, which included 466 patients with MFS, the median age of onset was 44 years old and age distribution showed two peaks at 30 to 39 and 50 to 59 years old. The other study showed that the median age of onset was 41 years old. Large epidemiological studies will help to estimate incidence more accurately in the future in old persons.

Several studies suggest the close correlation between anti-GQ1b IgG antibody and MFS or GBS with ophthalmoplegia These antibodies may participate in the development of ophthalmoplegia. Although we did not carry out anti-GQ1b IgG antibody tests in this patient because of technical problems, we think that the clinical manifestations and CSF albumincytological dissociation are enough to establish the diagnosis of MFS.

The differential diagnosis of MFS in the elderly people includes Bickerstaff’s brainstem encephalitis (BBE) and brain stem infarction. BBE is characterized by acute ophthalmoplegia, ataxia, hyperreflexia and disturbance of consciousness. The predominant clinical features in our patient, including ophthalmoplegia and ataxia, are consistent with the diagnosis of BBE. However, there were no consciousness disturbance or pyramidal signs found in our patient which made a differential diagnosis with BBE. Although the patient have cerebrovascular risk factors, such as age older than 65, hypertension and diabetes mellitus, brain stem infarction can be excluded by the absence of the infarction related symptoms and the brain MRI.

## References

[1] Mori M, Kuwabara S, Yuki N (2012). Fisher syndrome: clinical features, immunopathogenesis and management. Expert Rev Neurother.

[2] Odaka M, Yuki N, Hirata K (2001). Anti-GQ1b IgG antibody syndrome: clinical and immunological range. J Neurol Neurosurg Psychiatry.

[3] Lo YL, Ratnagopal P (2007). Cortico-hypoglossal and corticospinal conduction abnormality in Bickerstaff's brainstem encephalitis. Clin Neurol Neurosurg.

[4] Ito M, Kuwabara S, Odaka M, Misawa S, Koga M, Hirata K (2008). Bickerstaff's brainstem encephalitis and Fisher syndrome form a continuous spectrum: clinical analysis of 581 cases. J Neurol.

[5] Aranyi Z, Kovacs T, Sipos I, Bereczki D (2012). Miller Fisher syndrome: brief overview and update with a focus on electrophysiological findings. Eur J Neurol.

[6] Heckmann JG, Dutsch M (2012). Recurrent Miller Fisher syndrome: clinical and laboratory features. Eur J Neurol.

[7] Saito K, Shimizu F, Koga M, Sano Y, Tasaki A, Abe M (2013). Blood-brain barrier destruction determines Fisher/Bickerstaff clinical phenotypes: an in vitro study. J Neurol Neurosurg Psychiatry.

